# The relationship between the 3D electroanatomical mapping parameters of the left atrial posterior wall and the recurrence of paroxysmal atrial fibrillation

**DOI:** 10.3389/fcvm.2025.1522807

**Published:** 2025-02-14

**Authors:** Yuqiao Chen, Jun Zhuang, Xiaolong Li, Chunqin Zhang, Xinfu Cao, Zhiwei Xu, Xiu Feng

**Affiliations:** ^1^ Department of Cardiology, Changzhou Hospital of Traditional Chinese Medicine, Changzhou, Jiangsu, China; ^2^ Department of Cardiology, Anhui No.2 Provincial People’s Hospital, Hefei, Anhui, China; ^3^ Department of Echocardiography and Cardiology, The Third Affiliated Hospital of Soochow University, Changzhou, Jiangsu, China

**Keywords:** paroxysmal atrial fibrillation, left atrial posterior wall, 3D electroanatomical map, pulmonary vein isolation, recurrence

## Abstract

**Background:**

Pulmonary vein isolation (PVI) remains the cornerstone of catheter ablation in paroxysmal atrial fibrillation (PAF). However, the recurrence of AF after PVI needs further investigation. The left atrial posterior wall (LAPW) is embryologically related to the pulmonary vein and plays an important role in the initiation and maintenance of AF. This study aims to explore the relationship between the 3D electroanatomical mapping parameters of the LAPW and recurrence in patients with PAF.

**Methods:**

A retrospective analysis was conducted on patients with PAF who underwent PVI. Both clinical and procedural characteristics from the enrolled subjects were collected before PVI. 3D electroanatomical mapping anatomical and electrical parameters were measured and calculated in the CARTO system. Intergroup comparisons and multivariate logistic regression analysis were performed to demonstrate the relationship between the parameters of LAPW and AF recurrence. A combined prediction model for AF recurrence was constructed in this study.

**Results:**

A total of 120 patients were included in the final analysis. Among procedural characteristics, compared with Group 1 (no recurrence), there was a significantly larger posterior wall surface area (PWSA) (*p* = 0.013) and a percentage of very low-voltage area (PVLVA) (*p* < 0.001) in Group 2 (recurrence). Further analysis revealed that there was a significant difference between the two groups in terms of the distribution of VLVA (*p* = 0.026). Subsequently, in a multivariate logistic regression analysis, both PWSA and PVLVA were found to be independent risk factors for AF recurrence [odds ratio (OR): 1.457, 95% confidence interval (CI): 1.037–2.049, *p* = 0.030; OR: 1.059, 95% CI: 1.013–1.107, *p* = 0.012, respectively]. Finally, a prediction model that combined the PWSA with the PVLVA for AF recurrence was constructed to draw the receiver operating characteristic curve. The area under the curve of this model was 0.900 (0.827–0.973) (*p* < 0.001). The result, evaluated by using the Hosmer–Lemeshow goodness-of-fit test, showed that χ^2^ = 4.643 (*p* = 0.796).

**Conclusions:**

This study demonstrates that both PWSA and PVLVA were independent risk factors for AF recurrence. Moreover, we proposed a model that combined the PWSA with the PVLVA to predict the recurrence of AF, which may provide an approach for screening patients with PAF who may require attention for the LAPW.

## Introduction

Atrial fibrillation (AF) is an arrhythmia with increasing global incidence and prevalence (1, 2). AF is associated with serious adverse events and increased mortality (3–5). More and more evidence has shown that catheter ablation is an important approach for rhythm control in patients with paroxysmal AF (PAF) (6, 7), which also leads to a lower risk of adverse cardiovascular outcomes (8) and improvement in quality of life (9). Nowadays, pulmonary vein isolation (PVI) remains the cornerstone of catheter ablation in AF (7, 10). However, AF recurrence after catheter ablation remains a clinical concern (11, 12), which eventuates excess suffering in, and economic burden on, patients (13).

The left atrial (LA) posterior wall (PW) has been shown to contribute to the initiation and maintenance of AF (14, 15). As a consequence, ablation in the LAPW has evolved as a strategy in surgery (16). Notably, the LAPW and the esophagus are adjacent anatomically (16). This may be the leading lesion to the esophagus when catheter ablation, especially radiofrequency ablation (RFA), is performed in the LAPW. Disastrous consequences may occur when LA-esophageal fistula forms (17). Accordingly, the option of PW isolation should be exercised with caution (18).

Presently, research studies on LAPW ablation mostly focus on non-PAF (19, 20), but still traces of recurrence can be found in patients with PAF due to the triggering and maintenance mechanisms associated with the PW (21). It is worth studying how to identify these patients.

Currently, wide antral circumferential ablation of PVI is considered to be an effective measure to reduce AF recurrence (22), as the myocardium in the expansive area is thicker, resulting in a decrease in the first-pass isolation rate and an increase in the number of gaps (23). These are likely followed by a prolongation of operation time and an increase in complication rates. There is no unified standard ablation strategy for determining power, pressure, and duration time for PVI when targeted in the LAPW. The selection of a different ablation index (AI) would vary according to the degree of pain experienced by patients and concerns for the occurrence of LA-esophageal fistula. Hence, it is worth considering which patient is more suitable for wide antral ablation and for selecting an increased AI in the LAPW.

A three-dimensional (3D) electroanatomical model reconstructed by the CARTO-3 mapping system (version 6.0, Biosense Webster, Inc.) with non-fluoroscopy displays a very realistic model (24), which is in excellent concordance with CT (25). The area and the voltage of the LAPW can be determined in the CARTO system. This study aims to detect the relationship between the parameters of the LAPW in the 3D electroanatomical map and recurrence in patients with PAF.

## Methods

### Research subjects

A single-center, retrospective cohort method was performed in this study. Patients with PAF admitted to the Changzhou Hospital of Traditional Chinese Medicine from January 2021 to August 2023 and scheduled to undergo RFA were enrolled in this research. Inclusion criteria were as follows: (1) older than 18  years old; (2) diagnosis of PAF (defined as a sustained episode lasting <7 days or chemical and/or electrical cardioversion <7 days); (3) sinus rhythm when mapping; (4) patients who had received PVI alone; (5) complete basic information, procedure, and follow-up data. Exclusion criteria included: (1) severe organic heart disease (such as severe mitral regurgitation or stenosis, severe aortic regurgitation or stenosis, hypertrophic obstructive cardiomyopathy, etc.); (2) history of previous cardiac surgery; (3) patients with incomplete clinical and follow-up data. This study adhered to the Helsinki Declaration and received approval from the Scientific Ethics Committee of Changzhou Hospital of Traditional Chinese Medicine (2023-LL-017l); it was already registered in the Chinese Clinical Trial Registry (ChiCTR2300079137).

### Catheter ablation

Catheter ablation was carried out in the CARTO system. All procedures were performed without interruption of anticoagulant therapy and off antiarrhythmic medications. The high-density multielectrode mapping catheter [PentaRay (Biosense Webster, Inc.)] and irrigated contact force sensing ablation catheter [Thermocool SmartTouch ST or STSF (Biosense Webster, Inc.)] were introduced into the left atrium with transseptal puncture.

PentaRay reconstructed the electroanatomical map of the left atrium, including the main part of the left atrium, the left atrial appendage (LAA), and the pulmonary veins (PVs) after excluding the inspiratory phase through fast anatomical mapping (FAM). Then, ST or STSF examined the true boundary of the map further through contact force. Thus, a more realistic LA model could be revealed.

Ablation was performed with ST or STSF at a power of 35 W. The discharge time was determined according to the AI at each tag, with an intertag distance ≤6 mm. A target AI of 480–530 for the roof, anterior, and ridge; 420–450 for the inferior; and 360–380 for the posterior were recommended. PVI was defined by PV entrance and exit block.

### Clinical baseline indicators

Clinical baseline indicators of patients were collected, including gender, age, body mass index (BMI), duration of AF, CHA_2_DS_2_-VASc score, and medical history [including heart failure (HF), hypertension, diabetes mellitus (DM), and stroke or transient ischemic attack (TIA)].

Laboratory indicators [including urea nitrogen, creatinine, and N-terminal precursor protein brain natriuretic peptide (NT-proBNP)], and echocardiographic indicators [including the left atrial diameter (LAD), left ventricular end-diastolic diameter (LVEDD), left ventricular end-systolic diameter (LVESD), and left ventricular ejection fraction (LVEF)] were collected.

### Acquisition of anatomical and electrical parameters in the electroanatomical map

Anatomical and electrical parameters were measured in the 3D electroanatomical map of the CARTO system. All parameters were obtained from the maps before ablation.

**Figure 1:**
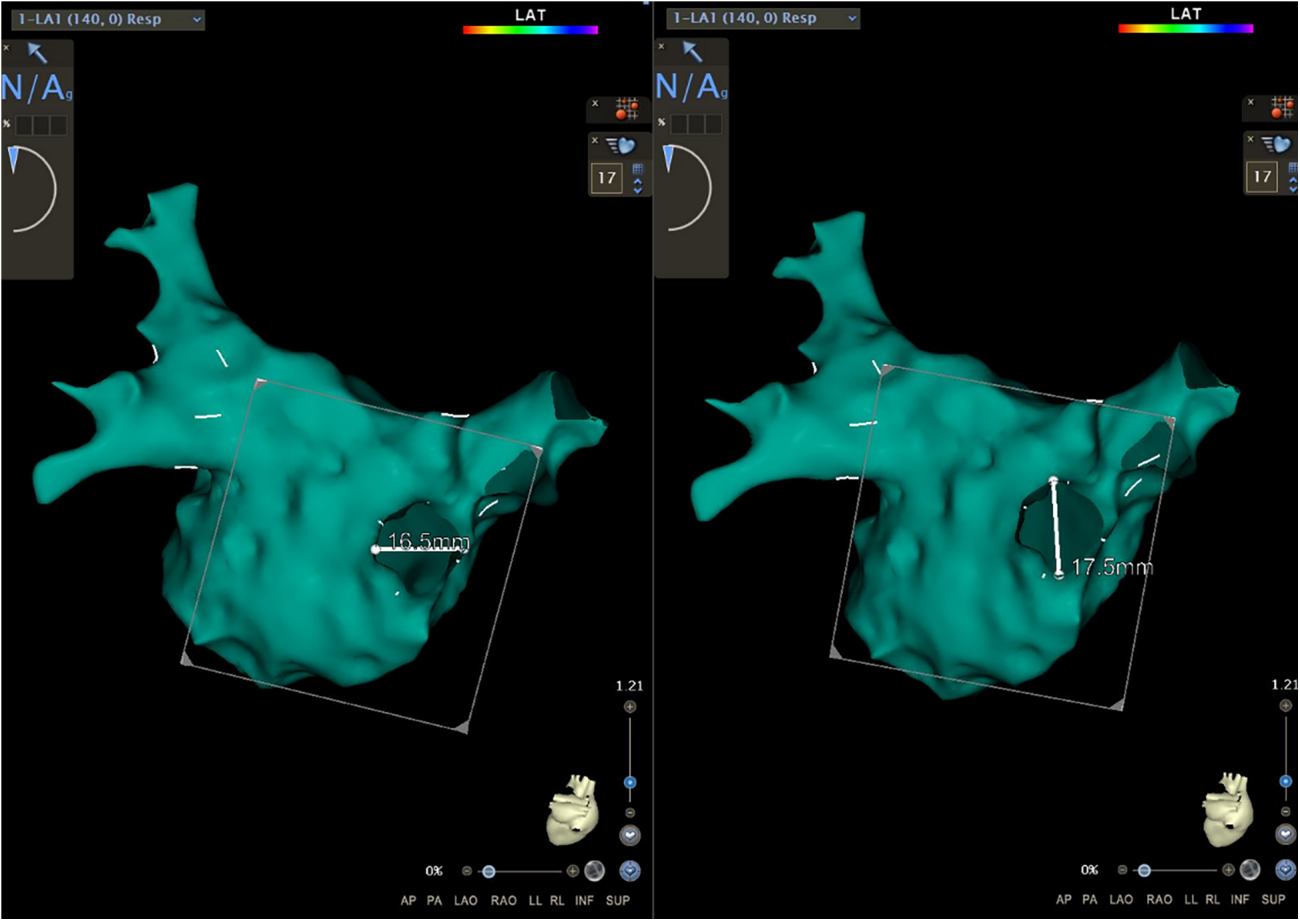
The function of the “clipping pane” was used to measure the diameters of the long axis and the short axis of each PV separately. RSPV, right superior pulmonary vein; RIPV, right inferior pulmonary vein; LSPV, left superior pulmonary vein; LIPV, left inferior pulmonary vein.

Because of the embryonic homology between the PW and the PVs, the area of the PV ostia was also measured in this study. A new map was saved (Map 1) after obtaining the initial map. In Map 1, we drew a short line along the axial direction of PVs from the superior, inferior, anterior, and posterior orientation to assist us in the next step (Figure 1). Then, we utilized the function of “clipping pane” to make a vertical section at the PV ostia [the right superior pulmonary vein (RSPV), the right inferior pulmonary vein (RIPV), the left superior pulmonary vein (LSPV), and the left inferior pulmonary vein (LIPV), respectively]. The measurement would be merged in the common pulmonary vein, and the accessory pulmonary vein was also measured if it resembled the main bifurcation. Subsequently, the diameters of the long axis (a) and the short axis (b) of each PV were measured separately (Figure 1). According to the calculation formula ellipse area = π × (a/2) × (b/2), the PV ostia area was obtained. The total area of the right PV (RPV) and left PV (LPV) was added up by each bifurcation. All parameters were measured independently by two researchers and an interobserver agreement was reached.

**Figure 2:**
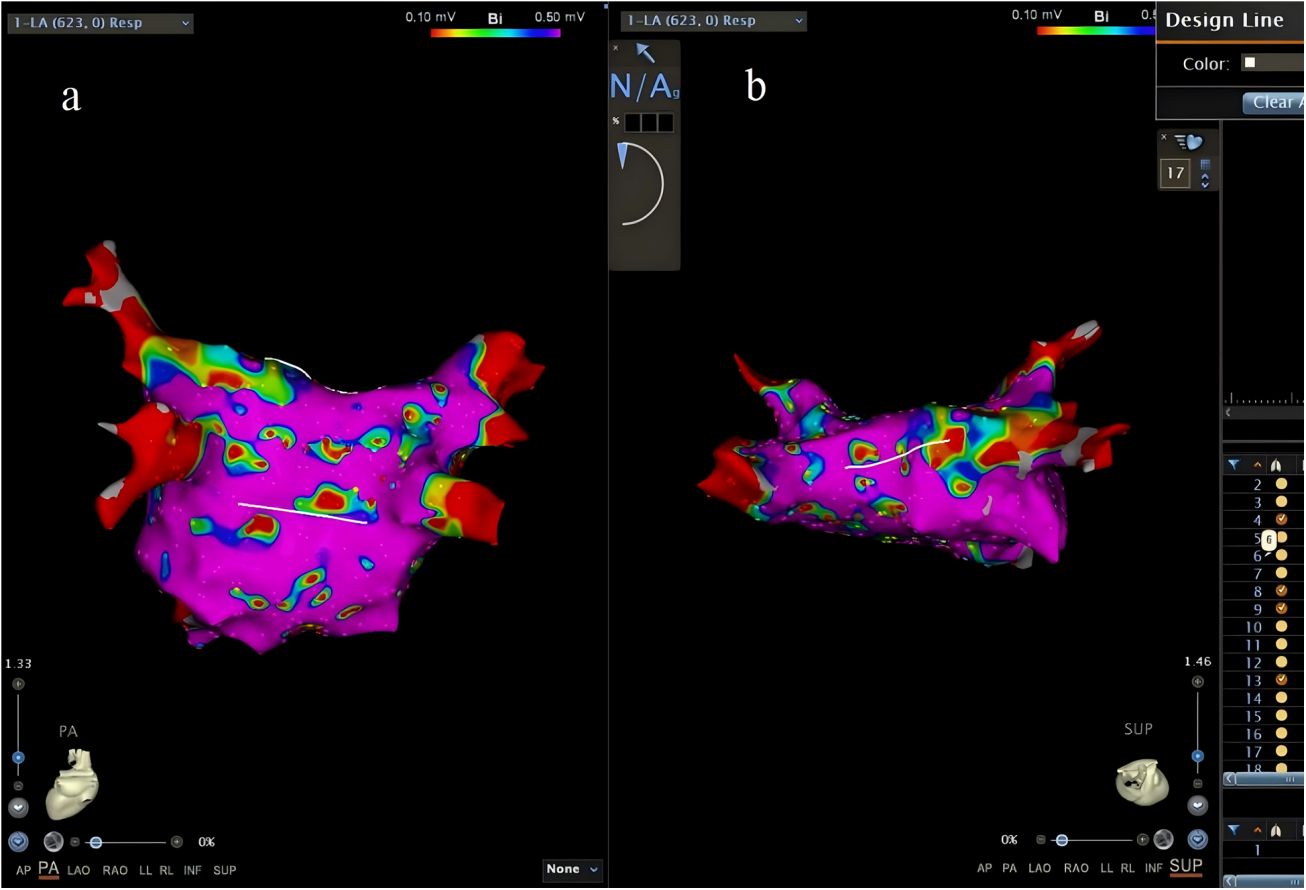
The inferior line (**a**) and the superior line (**b**) were drawn.

**Figure 3:**
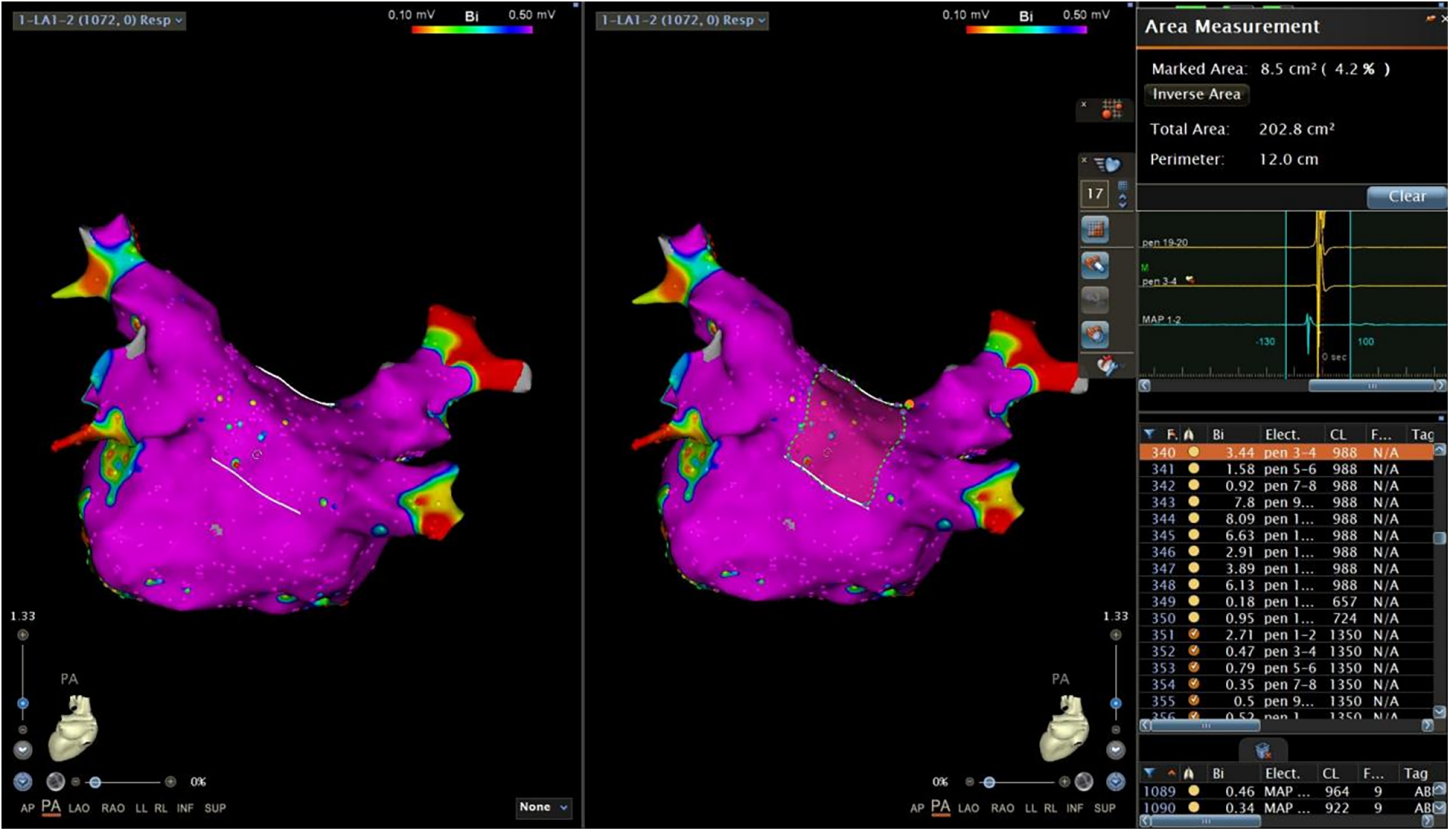
The function of “area measurement” was used to measure and calculate the PWSA. PWSA, posterior wall surface area.

A map was saved again (Map 2) after completing this step. In Map 2, we drew another short line at the roof and the bottom of the PW. The inferior line was defined as a line located at the lowest margin between the RIPV and the LIPV in the posterior view (Figure 2a). The superior line was defined as a line that connected the middle of the LSPV and RSPV ostia in the superior view (SUP) (Figure 2b). The PW surface area (PWSA) was composed of the area delineated by the superior and inferior line and 0.5–1 cm outside the PV ostia. Then, the function of “area measurement” was used to measure and calculate the area (Figure 3).

**Figure 4:**
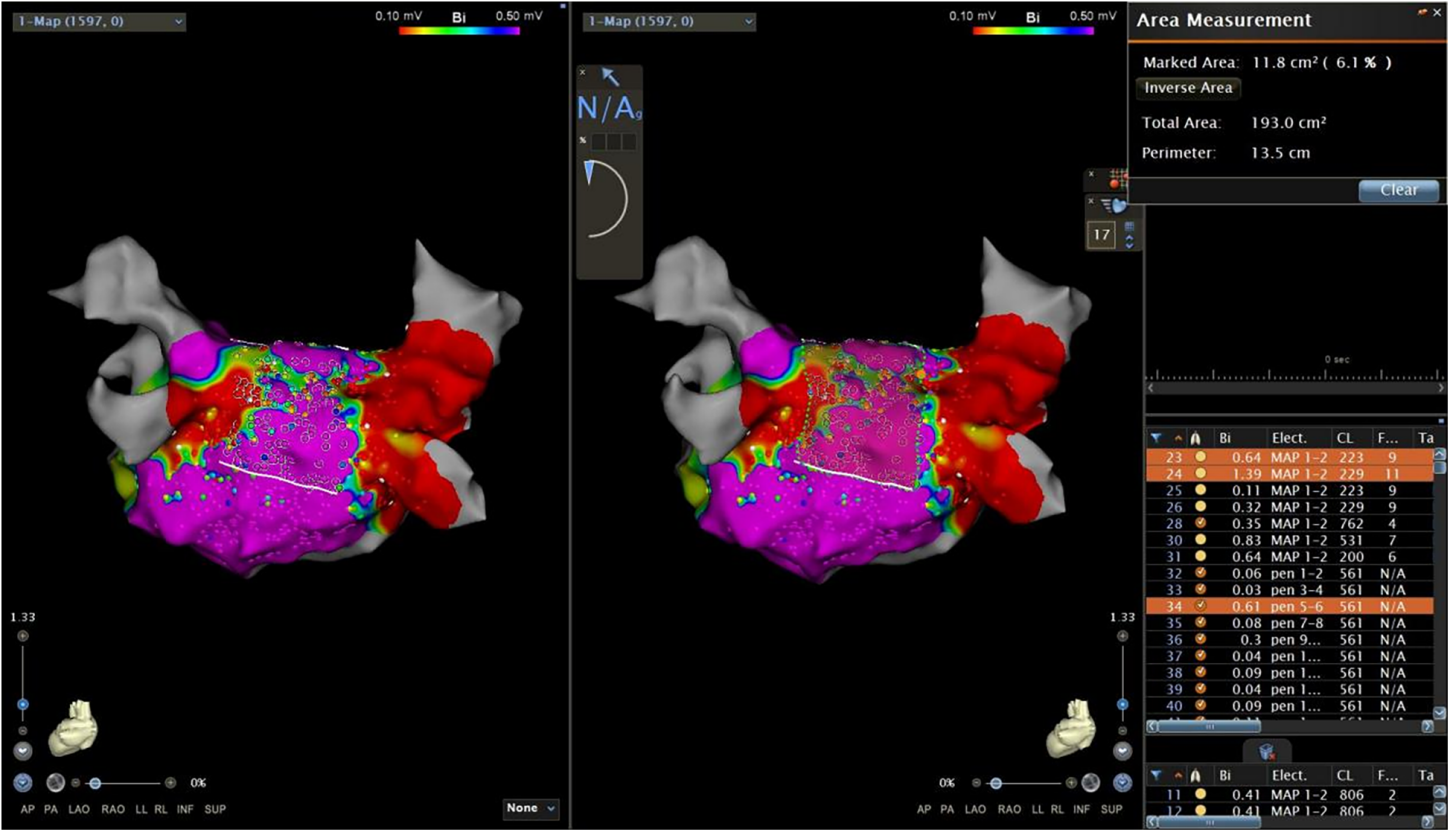
Copied activation mapping points in the PW area. PW, posterior wall.

**Figure 5:**
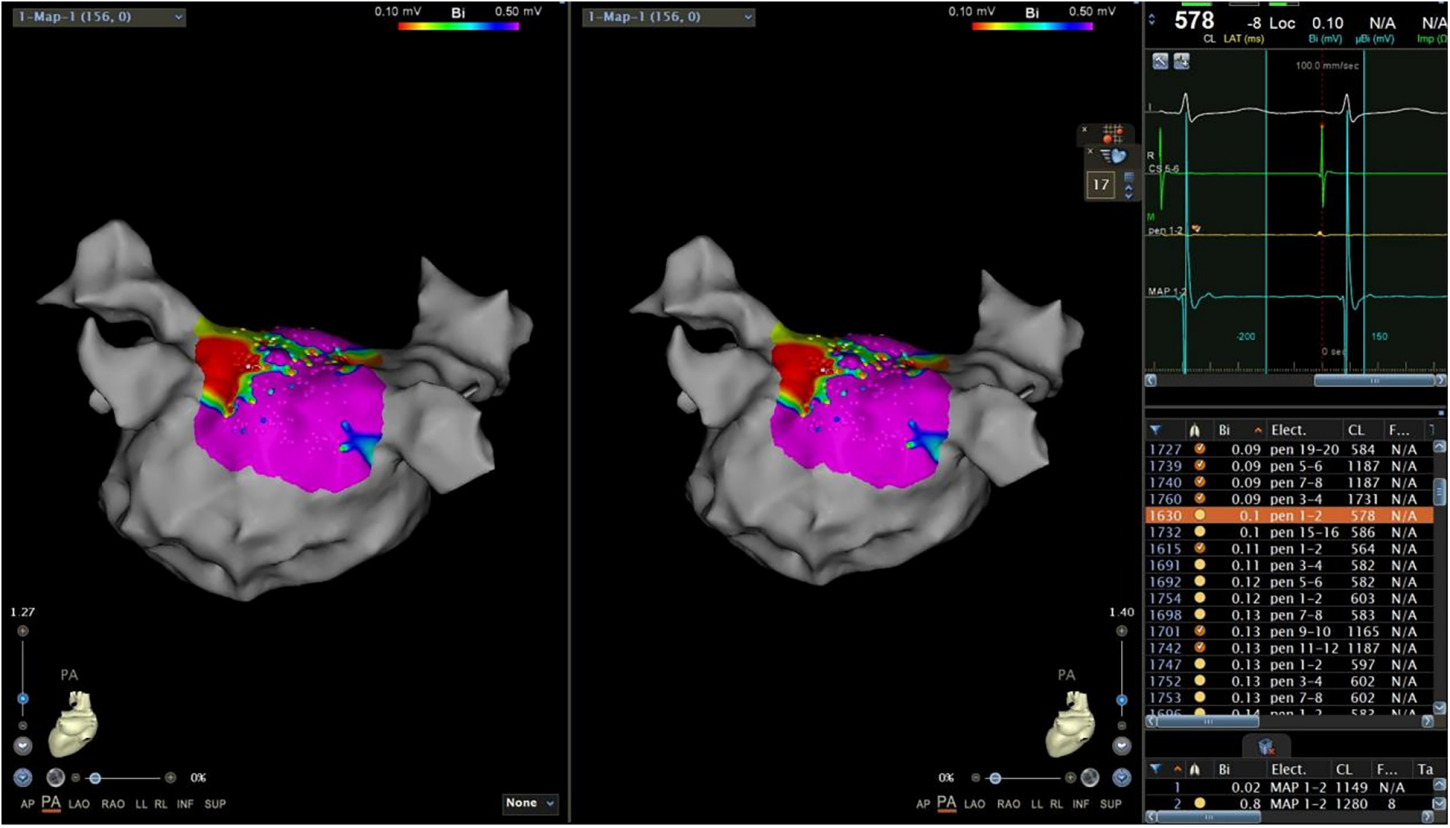
Copied points to another map and sorted all copied points by voltage.

The next step was to copy activation mapping points in the PW area to another map (Map 3) (Figure 4). In Map 3, after switching the bipolar map, we sorted all copied points by voltage (Figure 5). A very low-voltage area (VLVA) in the electrogram was defined as an amplitude of ≤0.1 mV. We subsequently counted the points with voltage ≤0.1 mV and calculated the percentage of the VLVA (PVLVA) in the LAPW (Figure 5)

**Figure 6:**
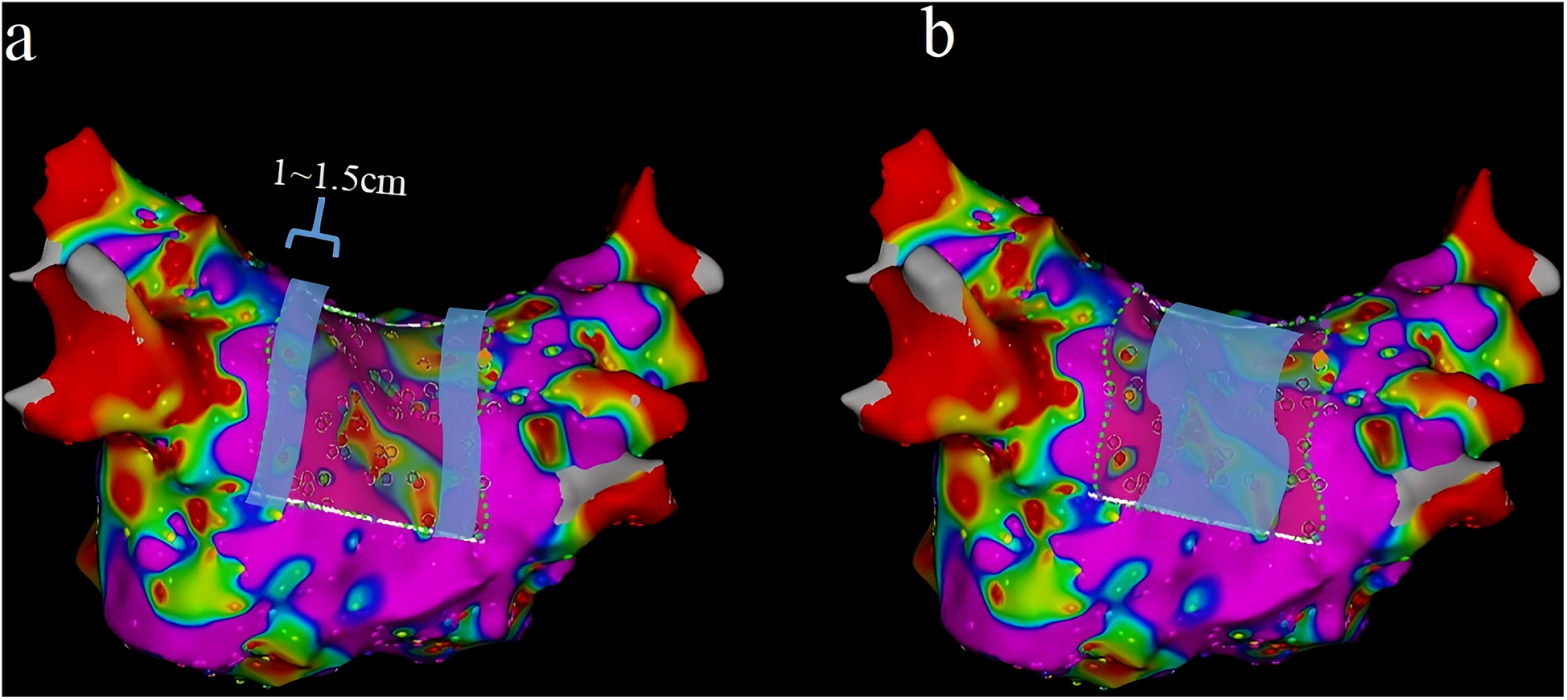
Two parts of the LAPW: PV ostium in the PW (the width was 1–1.5 cm) (**a**) and the intra-atrial region in the PW (**b**) LAPW, left atrial posterior wall; PV pulmonary veins.

In this study, we divided the distribution of the VLVA in the LAPW into two parts, namely, the connection of PVs and the LAPW (PV ostium in the PW) and the intra-atrial region in the PW (Figures 6a,b).

### Follow-up

Procedural success was defined as freedom from recurrent atrial arrhythmias lasting longer than 30 s after an initial 3-month blanking period. Recurrent arrhythmias were classified as being AF, atrial tachycardia, or both. Patients underwent a clinical review and 24 h Holter monitoring at 3, 6, and 12 months.

### Statistical analysis

Statistical analysis was performed using SPSS Statistics Software version 26 (IBM, Inc.). Measurement data that followed a normal distribution pattern are expressed as mean ± standard deviation (x ± s). In cases where these data had a mildly skewed distribution, the median was used. Either an independent sample *t*-test or the Kruskal–Wallis test was performed in intergroup comparisons. Counting data were presented as a percentage, and intergroup comparisons were conducted using the chi-square test or Fisher's precision probability test. A univariate logistic regression analysis assessed the association of different factors and AF recurrence. After collinearity analysis (tolerance <0.1 or variance inflation factor >10 meant multicollinearity between variables), a multivariate logistic regression analysis was employed to evaluate which factor was an independent risk factor for AF recurrence. A *p*-value <0.05 was considered statistically significant. If a covariate that changed the estimates of AF recurrence had a significant association (*p* < 0.10) in the univariate logistic regression analysis, it would be included as a potential confounding factor in the multivariate logistic regression analysis. A receiver operating characteristic (ROC) curve was used to construct the combined prediction model for AF recurrence. If the area under the curve (AUC) was greater than 0.75, it indicated better predictive ability. A Hosmer–Lemeshow goodness-of-fit test was performed to evaluate the calibration ability of the prediction model, and a *p*-value >0.05 was considered a good fit of the model.

## Results

### Baseline characteristics

**Figure 7:**
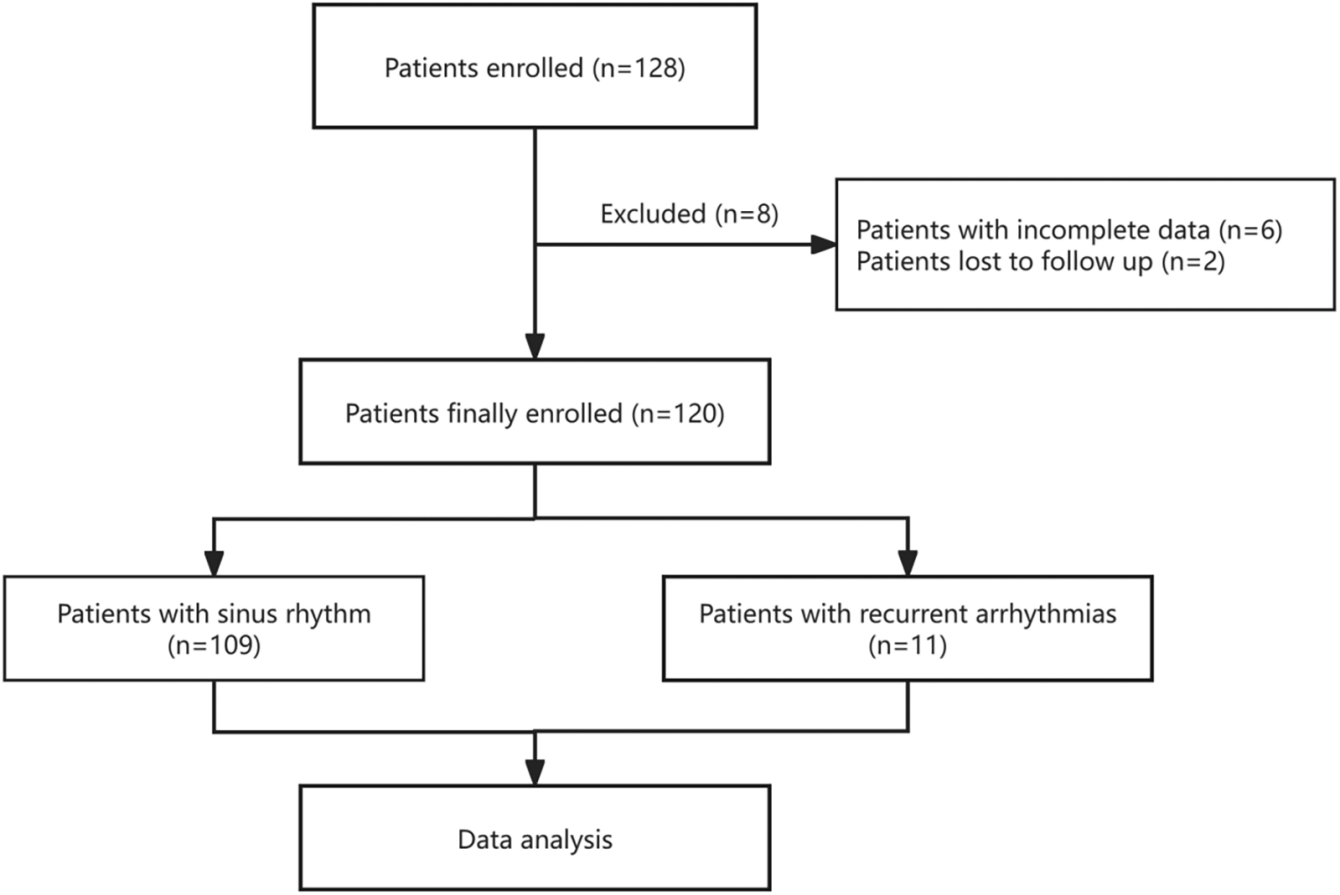
Flowchart of the study.

According to the inclusion and exclusion criteria, a total of 128 patients were enrolled in this study. Of the initial participants, eight were excluded from the final analysis (including six incomplete data and two lost to follow-up) (Figure 7). Consequently, the baseline characteristics of 120 patients are presented in Table 1. According to the occurrence or non-occurrence of recurrent arrhythmias, these patients were divided into Group 1 (no recurrence) and Group 2 (recurrence). Group 1 comprised 49 males and 60 females, averaging 67.66 ± 12.60 years. Group 2 was composed of four males and seven females, averaging 67.18 ± 13.23 years. There was no significant difference between the two groups in terms of gender, age, BMI, duration of AF, CHA_2_DS_2_-VASc score, history of hypertension, DM and stroke, or TIA, except for a history of HF (*p* = 0.028). There was also no statistical difference in terms of urea nitrogen, creatinine, NT-proBNP, LAD, LVEDD, LVESD, and LVEF (Table 1).

**Table 1:** Baseline characteristics.

	Group 1 (no recurrence)	Group 2 (recurrence)	*p*-value
*N* (cases)	109	11	
Female (%)	60 (55.0)	7 (63.6)	0.585
Age (years)	67.66 ± 12.60	67.18 ± 13.23	0.905
BMI (kg/m^2^)	23.97 ± 3.19	22.16 ± 5.03	0.094
Duration of AF (months)	6 (1–36)	12 (2–72)	0.065
CHA_2_DS_2_-VASc			0.270
0 (%)	13 (11.9)	1 (9.1)	
1 (%)	19 (17.4)	1 (9.1)	
≥2 (%)	77 (70.6)	9 (81.8)	
Comorbidity			
HF, *n* (%)	26 (23.9)	6 (54.5)	0.028*
Hypertension, *n* (%)	63 (57.8)	8 (72.7)	0.337
DM, *n* (%)	25 (22.9)	2 (18.2)	0.719
Stroke or TIA, *n* (%)	7 (6.4)	0 (0)	0.386
Laboratory indicators			
Urea nitrogen (mmol/L)	6.32 ± 2.03	6.68 ± 2.47	0.586
Creatinine (μmol/L)	71.24 ± 21.87	68.30 ± 18.68	0.668
NT-proBNP (ng/ml)	420.06 (192.31–900.13)	1,033.90 (226.30–3,463.10)	0.063
Echocardiographic indicators			
LAD (mm)	40.28 ± 4.84	41.18 ± 5.93	0.567
LVEDD (mm)	47.91 ± 5.00	46.55 ± 4.08	0.384
LVESD (mm)	31.53 ± 5.50	30.91 ± 4.28	0.716
LVEF (%)	62.44 ± 8.56	60.64 ± 5.80	0.496

BMI, body mass index; AF, atrial fibrillation; CHA_2_DS_2_-VASc, stroke risk score of AF patients; HF, heart failure; DM, diabetes mellitus; TIA, transient ischemic attack; NT-proBNP, N-terminal precursor protein brain natriuretic peptide; LAD, left atrial diameter; LVEDD, left ventricular end-diastolic diameter; LVESD, left ventricular end-systolic diameter; LVEF, left ventricular ejection fraction.

* *p*-value < 0.05.

### Procedural characteristics

Procedural characteristics are presented in Table 2. The areas of the PV ostia (RPV and LPV, respectively) and the PWSA and the PVLVA were measured and calculated. The complications were recorded. Among the procedural characteristics, patients in Group 2 were found to have a significantly larger PWSA (*p* = 0.013) and PVLVA (*p* < 0.001) (Table 2).

**Table 2:** Procedural characteristics.

	Group 1 (no recurrence)	Group 2 (recurrence)	*p*-value
RPV ostia area (cm^2^)	4.90 ± 1.61	5.68 ± 2.20	0.144
LPV ostia area (cm^2^)	3.11 ± 1.30	3.31 ± 0.85	0.636
PWSA (cm^2^)	10.13 ± 2.51	12.13 ± 2.48	0.013*
PVLVA (%)	0.00 (0.00–4.12)	11.56 (0.00–35.21)	<0.001*
Complications (%)	6.42	9.09	0.735

RPV, right pulmonary vein; LPV, left pulmonary vein; PWSA, posterior wall surface area; PVLVA, percentage of a very low-voltage area.

* *p*-value < 0.05.

### Analysis of the distribution of the VLVA

In patients with the VLVA in the LAPW, the proportions of distribution of the VLVA in the PV ostium in the PW, the intra-atrial region in the PW, and the mixed areas (contained both) were 60.0%, 20.0%, and 20.0%, respectively, in Group 1 and 12.5%, 12.5%, and 75.0%, respectively, in Group 2 (*p* = 0.026) (Table 3).

**Table 3:** Analysis of the distribution of the VLVA.

	Group 1 (no recurrence)	Group 2 (recurrence)	*p*-value
VLVA in the PV ostium (%)	60.0	12.5	
VLVA in the intra-atrial region (%)	20.0	12.5	
VLVA in the mixed area (%)	20.0	75.0	
Fisher's precision probability test			0.026*

VLVA, very low-voltage area; PVs, pulmonary veins.

* *p*-value < 0.05.

### Univariate logistic regression analysis of the baseline and procedural characteristics with AF recurrence

AF recurrence was used as the dependent variable and baseline and procedural characteristics as independent variables. A univariate logistic regression analysis was performed to identify the potential factors associated with AF recurrence. The result showed that the BMI [odds ratio (OR): 0.839, 95% confidence interval (CI): 0.682–1.032, *p* = 0.096], HF (OR: 3.831, 95% CI: 1.080–13.585, *p* = 0.038), PWSA (OR: 1.369, 95% CI: 1.056–1.775, *p* = 0.018), and PVLVA (OR: 1.064, 95% CI: 1.025–1.103, *p* = 0.001) were potential factors related to AF recurrence (*p* < 0.10) (Table 4).

**Table 4:** Univariate logistic regression analysis of baseline and procedural characteristics with AF recurrence.

Covariate	OR	*p*-value
Female, *n* (%)	1.429 (0.395–5.167)	0.586
Age (years)	0.997 (0.950–1.047)	0.904
BMI (kg/m^2^)	0.839 (0.682–1.032)	0.096*
Duration of AF (months)	1.003 (0.995–1.010)	0.471
CHA_2_DS_2_-VASc	1.143 (0.803–1.629)	0.458
HF, *n* (%)	3.831 (1.080–13.585)	0.038*
Hypertension, *n* (%)	1.947 (0.490–7.742)	0.344
DM, *n* (%)	0.747 (0.151–3.683)	0.720
LAD (mm)	1.039 (0.913–1.182)	0.564
RPV ostia area (cm^2^)	1.003 (0.999–1.006)	0.146
LPV ostia area (cm^2^)	1.001 (0.997–1.006)	0.643
PWSA (cm^2^)	1.369 (1.056–1.775)	0.018*
PVLVA (%)	1.064 (1.025–1.103)	0.001*

BMI, body mass index; AF, atrial fibrillation; CHA_2_DS_2_-VASc, stroke risk score of AF patients; HF, heart failure; DM, diabetes mellitus; LAD, left atrial diameter; RPV, right pulmonary vein; LPV, left pulmonary vein; PWSA, posterior wall surface area; PVLVA, percentage of very low-voltage area.

* *p*-value < 0.10.

### Multivariate logistic regression analysis of potential factors on AF recurrence

Although the *p*-value of the LAD was >0.10 in the univariate logistic regression analysis, it was a common influencing clinical factor, which was also included in the multivariate logistic regression analysis. The collinearity analysis of the BMI, HF, LAD, PWSA, and PVLVA were performed at first. The results showed that there was no linear relationship between these variables (Table 5). Then, a multivariate logistic regression analysis of potential factors demonstrated that both the PWSA and the PVLVA were independent risk factors for AF recurrence (OR: 1.457, 95% CI: 1.037–2.049, *p* = 0.030; OR: 1.059, 95% CI: 1.013–1.107, *p* = 0.012, respectively) (Table 6).

**Table 5:** Collinearity analysis.

Variables	Tolerance	VIF
BMI (kg/m^2^)	0.751	1.332
HF, *n* (%)	0.723	1.382
LAD (mm)	0.591	1.693
PWSA (cm^2^)	0.832	1.201
PVLVA (%)	0.879	1.138

BMI, body mass index; HF, heart failure; LAD, left atrial diameter; PWSA, posterior wall surface area; PVLVA, percentage of very low-voltage area.

Tolerance < 0.1 or VIF > 10 meant multicollinearity between variables.

**Table 6:** Multivariate logistic regression analysis of potential factors for AF recurrence.

Variables	OR	*p*-value
BMI (kg/m^2^)	0.848 (0.648–1.110)	0.230
HF, *n* (%)	2.953 (0.478–18.235)	0.244
LAD (mm)	0.897 (0.725–1.100)	0.318
PWSA (cm^2^)	1.457 (1.037–2.049)	0.030*
PVLVA (%)	1.059 (1.013–1.107)	0.012*

BMI, body mass index; HF, heart failure; LAD, left atrial diameter; PWSA, posterior wall surface area; PVLVA, percentage of very low-voltage area.

* *p*-value < 0.05.

### ROC curve and curve fitting

**Figure 8:**
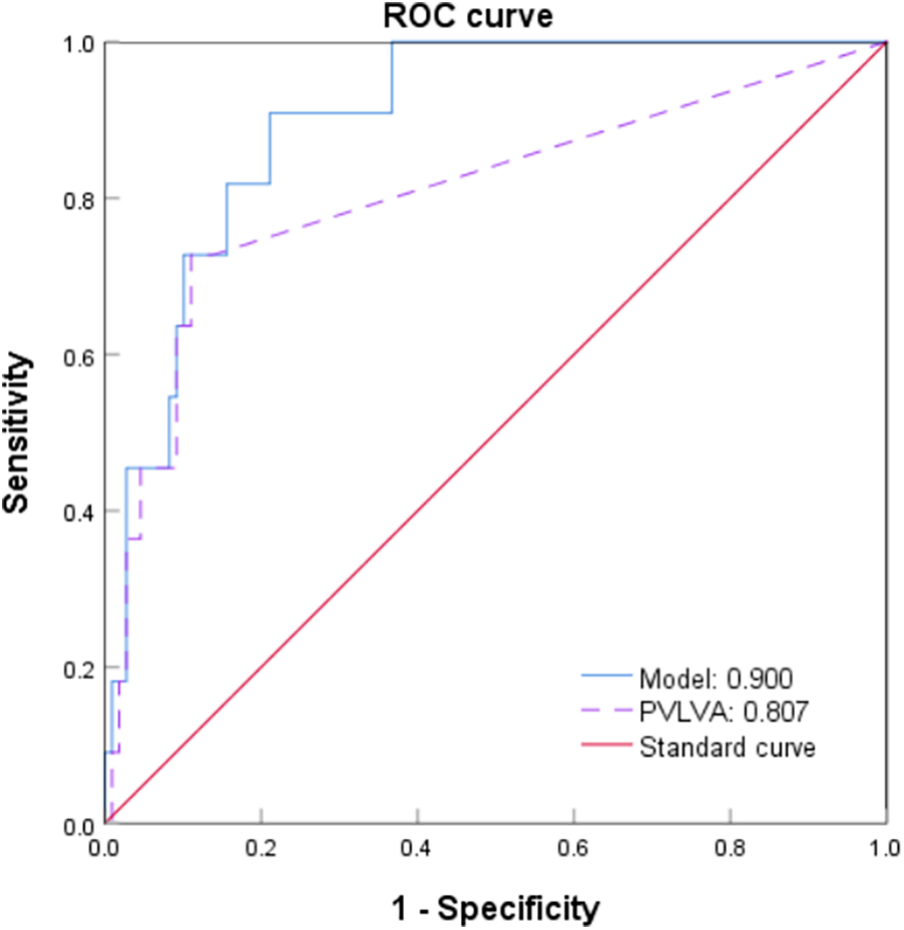
ROC curve of a combined model for predicting AF recurrence. ROC, receiver operating characteristic; AF, atrial fibrillation.

**Figure 9:**
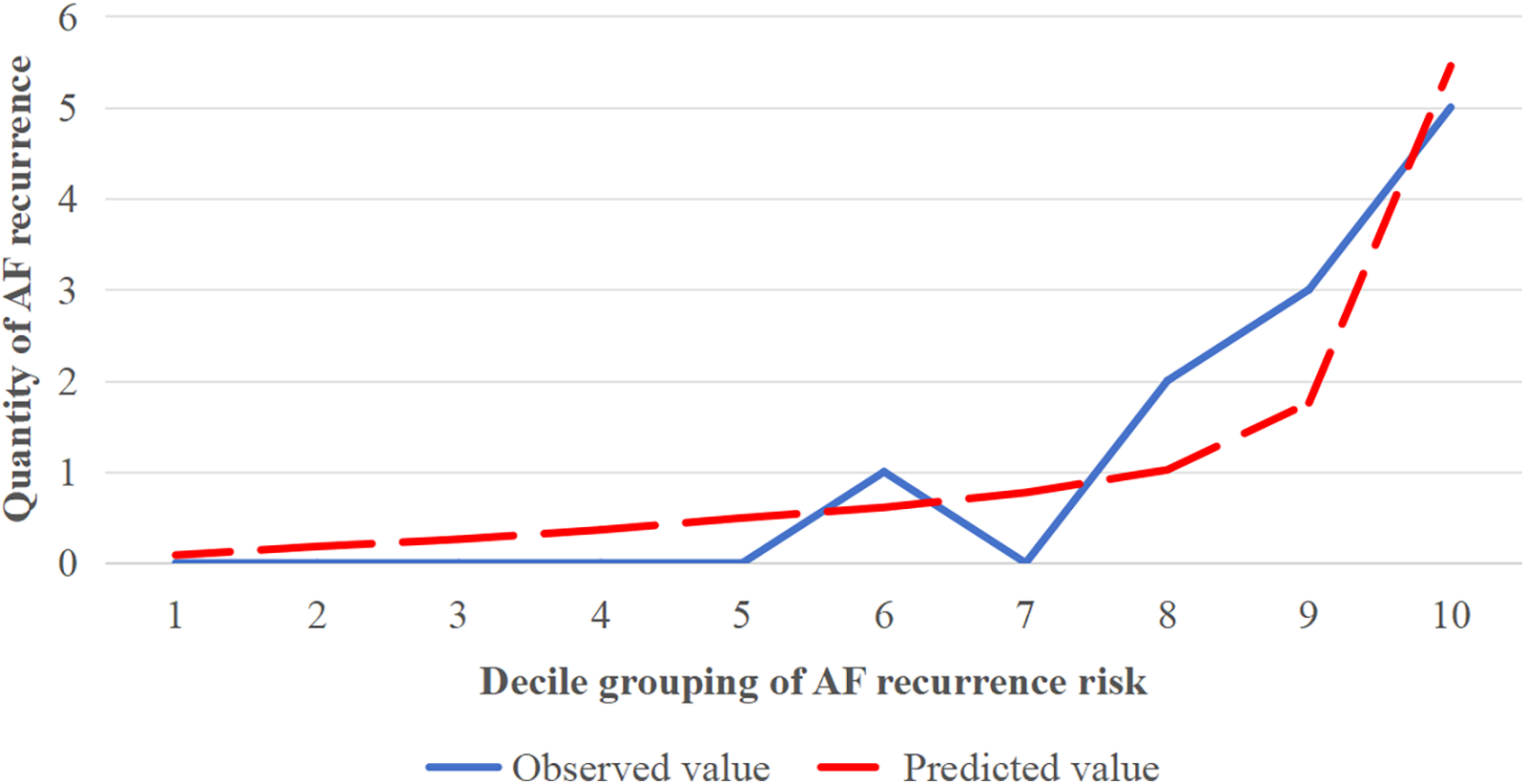
Hosmer–Lemeshow goodness-of-fit test (curve fitting).

According to the logistic regression analysis of this study, the HF, LAD, PWSA, and PVLVA were included to construct the combined prediction model for AF recurrence and to draw the ROC curve (Figure 8). The equation was Logit (*P*) = −0.057 + 1.296 × HF − 0.175 × LAD + 0.342 × PWSA + 0.066 × PVLVA. The finding revealed that the AUC of this model was 0.900 (0.827–0.973) (*p* < 0.001) (Figure 8). The Hosmer–Lemeshow goodness-of-fit test was performed to evaluate the calibration ability of the prediction model (Figure 9). The result suggested that there was no statistical difference between the predicted value of the model and the observed value (*χ*^2^ = 4.643, *p* = 0.796).

## Discussion

The main findings of this study revealed that for patients with PAF who underwent PVI potential risk factors for AF recurrence could be discovered through the 3D electroanatomical map in the CARTO system. The area of the LAPW and the PVLVA may be associated with PAF recurrence.

Spontaneous trigger activity and rotors in the LAPW have been reported previously (26). In structurally normal hearts, focal discharges from the PVs and the LAPW are important initiating factors of AF (27). Recurrent PAF after one year is partly due to LAPW triggers (28). For patients with PAF who underwent PVI alone, the recurrence was observed to involve the reentry of PVs and non-PV triggers. The recovery of PV ostium potential in the PW and other triggers within the intra-atrial region in the PW that were not ablated are recurrent factors associated with the LAPW. This may be elucidated from an anatomical standpoint. The LAPW is embryologically related to the PV (29). There is some interplay between the PVs and the LAPW musculature (30), resulting in conduction delay histopathologically and functionally (31). Non-contact mapping has also demonstrated significant conduction abnormalities in the LAPW in patients with PAF during sinus rhythm (32). Consequently, a part of recurrent foci of patients with PAF are derived from the LAPW, which is supported by numerous anatomical and electrical foundations.

Currently, research on LAPW triggers is primarily focused on non-PAF, with the most common intervention method being PW isolation (such as endocardial box isolation) (18, 19). There are only a few studies focusing on the PW in PAF. In this study, we measured the PWSA and the VLVA of patients with PAF before PVI in the CARTO system. The result demonstrated that there was a wider PWSA (*p* = 0.013) in recurrent patients. The PWSA was an independent risk factor for AF recurrence (OR: 1.457, 95% CI: 1.037–2.049, *p* = 0.030). However, there was no significant difference between the two groups in the LAD and the area of PV ostia. The lack of significant differences in LA size and AF burden among these patients may account for these results. In addition, the mild degree of LA structural remodeling may contribute to the outcomes. This indicated that the increase in the PWSA may not be due to the overall expansion of the left atrium. As AF persists and progresses, there would be a gradual expansion in both LA volume and PV opening area. The larger the LAPW, the broader the range of heterogeneity that can potentially occur, thereby increasing the likelihood of triggering foci in the PW.

The magnitude and conduction time of the electrical potential serve as indicators for cardiomyocyte health (33). Atrial fibrosis can impact the extracellular matrix, thereby influencing both electrical and mechanical functioning of the LA (34). The extent of atrial fibrosis is closely linked to AF recurrence (35, 36). Low-voltage or non-voltage electrograms and slow conduction are often considered manifestations of scar tissue or fibrosis (33, 37). The normal range of bipolar voltage of the LA is usually 0.3–1.0 mV (38). The region where a voltage <0.5 mV is typically considered is the low-voltage zone (39). Therefore, we defined the region with amplitude ≤0.1 mV as the VLVA in this study. The result showed that the recurrence group had a greater range of VLVA (*p* < 0.001). The PVLVA was also an independent risk factor for AF recurrence (OR: 1.059, 95% CI: 1.013–1.107, *p* = 0.012). Compared with Group 1, the VLVA of Group 2 exhibited a more extensive distribution, primarily within the mixed area (*p* = 0.026). This observation may suggest an increased presence of potential triggering foci in the PW or a greater complexity in heterogeneity at the junction between the PW and the PVs in these patients. The complexity may also include a significant anisotropy of crista terminalis and the subendocardial fiber around the septopulmonary bundle (30, 32). Anatomically, certain patients exhibit a layer of adipose tissue that separates the septopulmonary bundle from the atrial myocardium, which impedes the penetration of radiofrequency energy. Consequently, this barrier allows for the persistence of epicardial conduction through the septopulmonary bundle, preventing the achievement of a true conduction block, which related to AF recurrences (40). Because of the early stage of AF, most patients with PAF exhibit a relatively healthy myocardium, with fewer conduction abnormalities or low-voltage areas. This underscores the significance of early detection of abnormal myocardial electrical activity.

Although there are many prediction models, the accuracy of predicting the recurrence of AF was not high enough (41). These prediction models predominantly include variables such as age, BMI, category of AF, LAD, LVEF, current smoking habits, and estimated glomerular filtration rate. The majority of these models have an AUC below 0.75. One potential explanation for this could be that these variables are almost entirely derived from non-invasive evaluations of the patient, with limited data on the electrical and anatomical characteristics of the patient's atria, which are frequently associated with AF recurrence. In this study, the prediction model that the PWSA combined with the PVLVA for AF recurrence was constructed by potential clinical factors and prior analysis. The AUC of the model achieved a moderate efficacy of 0.900 (0.827–0.973) (*p* < 0.001) in predicting AF recurrence after RFA, which was verified by using the Hosmer–Lemeshow goodness-of-fit test (*p* > 0.05). The advantage of this predictive model lay in its specific focus on the LAPW, encompassing both PV ostium in the PW and the intra-atrial region in the PW, while simultaneously considering the area of the PW and the degree and distribution of abnormal conduction.

At present, ongoing efforts are focused on achieving permanent PVI by techniques during an initial RFA (42). However, given the anatomical and electrophysiological significance of the LAPW, as well as the potential complications associated with inappropriate ablation, determining the optimal ablation depth for this region during PVI is a question worthy of careful consideration. The latest study indicated that during ablation in the LAPW, selecting the strategy of 50 W/>10–15 s, as opposed to 40 W/10–15 s and 90 W/4 s, facilitated a more effective transmural lesion and resulted in a more durable block (43). Furthermore, for patients with PAF exhibiting an abnormal PW matrix, selecting ablation strategies for other regions of the PW beyond PVI also warrants further investigation.

## Limitations

There were several limitations in this study. First of all, this study was a single-center, retrospective cohort study, with a relatively small sample size. Second, there was still a possibility of residual confounding factors in the multivariate logistic regression model. Moreover, although activation mapping points were considered as uniformly as possible on the map, it still could not completely represent the real situation of voltage distribution. Finally, the mechanism between the PWSA and the PVLVA with AF recurrence is currently unknown. Therefore, more research is needed to clarify the specific pathophysiological mechanism.

## Conclusions

In summary, this study provided a method for predicting recurrence in patients with PAF, utilizing parameters related to the LAPW based on the CARTO system, prior to undergoing PVI. The analysis demonstrated that both PWSA and PVLVA were independent risk factors for AF recurrence. Moreover, we proposed a model that combined the PWSA with the PVLVA to predict the recurrence of AF, which may provide an approach for screening PAF patients who may require attention for the LAPW.

## Data Availability

The raw data supporting the conclusions of this article will be made available by the authors, without undue reservation.
